# A bibliometric analysis of apoptosis in glaucoma

**DOI:** 10.3389/fnins.2023.1105158

**Published:** 2023-02-06

**Authors:** Jia-Heng Zhang, Mei-Juan Wang, Ya-Ting Tan, Jia Luo, Shu-Chao Wang

**Affiliations:** ^1^Center for Medical Research, The Second Xiangya Hospital of Central South University, Changsha, Hunan, China; ^2^Clinical Medicine 5-Year Program, 19 Grade, Xiangya School of Medicine, Central South University, Changsha, China; ^3^Medical Imaging Center, Qingdao West Coast New District People's Hospital, Qingdao, Shandong, China; ^4^Department of Anatomy and Neurobiology, School of Basic Medical Sciences, Central South University, Changsha, Hunan, China; ^5^Hunan Key Laboratory of the Research and Development of Novel Pharmaceutical Preparations, Changsha Medical University, Changsha, China; ^6^National Clinical Research Center for Mental Disorders, The Second Xiangya Hospital of Central South University, Changsha, China

**Keywords:** bibliometric, CiteSpace, VOSviewer, apoptosis, glaucoma, retinal ganglion cell

## Abstract

**Background:**

Glaucoma is the first irreversible and second blindness disease, which is characterized by the death of retinal ganglion cells (RGCs) and degeneration of the optic nerve. Previous works have indicated that apoptosis is the main reason for RGC death in glaucoma. Although many studies have investigated the mechanism of apoptosis and different strategies targeting apoptosis to protect the RGCs and finally recover the impaired vision in the glaucoma. However, the global trend and hotspots of apoptosis in glaucoma have not been well illustrated and discussed.

**Methods:**

Documents were extracted from the Web of Science Core Collection on November 2, 2022. We selected articles and reviews published in English from January 1, 1999 to November 1, 2022 to perform visual analysis and statistical analysis of countries, institutions, authors, references and keywords by VOSviewer 1.6.18 and CiteSpace 5.8.

**Results:**

The publications about apoptosis in glaucoma show an increasing trend over time. Besides, the authors, institutions in the US and China published the most numbers of articles with the highest citation, which may be leading the research in the field of apoptosis in glaucoma. Last, series of advanced research results, technology and treatment for glaucoma, such as the discovery of key regulatory mechanisms on RGC apoptosis are emerging and will provide precise strategies for the treatment of glaucoma.

**Conclusion:**

This research will broaden our comprehension about the role of apoptosis in the process of glaucoma, and provide guidelines for us in basic research and disease treatment in the further.

## Introduction

Glaucoma affects 80 million people worldwide (Ju et al., 2022; Liu et al., 2022). It is characterized by the death of retinal ganglion cells (RGCs) and the degeneration of the optic nerve, ultimately leading to progressively impaired vision (Wang et al., 2017a; Chen et al., 2022b). High intraocular pressure (IOP) is thought to be the primary risk factor for RGC death (Hu et al., 2020; Ju et al., 2022). Previous studies have indicated that apoptosis is the main reason for RGC loss in glaucoma (Hu et al., 2021; Cordeiro et al., 2022). Apoptosis is a type of programmed cell death that usually occurs in developing tissues and during homeostasis in organisms (Wang et al., 2019; Yan et al., 2022, 2023). Apoptosis is characterized by chromatin condensation, nuclear fragmentation, and the formation of apoptotic bodies (Zhang et al., 2021; Wang et al., 2023). In recent decades, many studies have indicated that RGC apoptosis occurs in glaucoma, and the mechanisms of RGC apoptosis have been widely investigated (Hu et al., 2021; Cordeiro et al., 2022). In addition, different strategies targeting apoptosis to protect RGCs and recover impaired vision in glaucoma are emerging (Chitranshi et al., 2018; Cordeiro et al., 2022). However, the global trends and research hotspots of apoptosis in glaucoma have not been well illustrated and discussed.

Compared to a review in general, bibliometric analysis is a mathematically and statistically feasible discipline to summarize and predict current and future research hotspots and trends by evaluating specific research areas based on main authors, research institutes, journals, citation frequency, keywords, etc (Hu et al., 2022; Zhang et al., 2022). CiteSpace and VOSviewer are two common and important bibliometric analysis software (Chen et al., 2022a; Zhang et al., 2022; Zhao et al., 2022). By using them alone or in combination, we obtain a better analysis of research trends and hotspots according to different specifications (Romero and Portillo-Salido, 2019; Zhao et al., 2022).

Our group focused on the research of apoptosis and necroptosis in the nervous system, which is involved in glaucoma and trauma. A bibliometric analysis broadens our understanding of existing research hotspots, future trends, and implications of apoptosis in glaucoma and other diseases, which can help to clarify the research status and identify the latest and most influential progress. In addition, a bibliometric analysis may provide us with useful tools to make clinical decisions and propose guidelines by analyzing keywords about drugs, examination, and treatment (Dai et al., 2022).

In this current study, we aimed to summarize and analyze the current research hotspots and future research trends of apoptosis in glaucoma using bibliometric analysis. This study deepens our understanding of the role of apoptosis in glaucoma and provides guidelines for basic research and disease treatment in the future.

## Materials and methods

### Data collection and strategy for data retrieval

**Figure 1:**
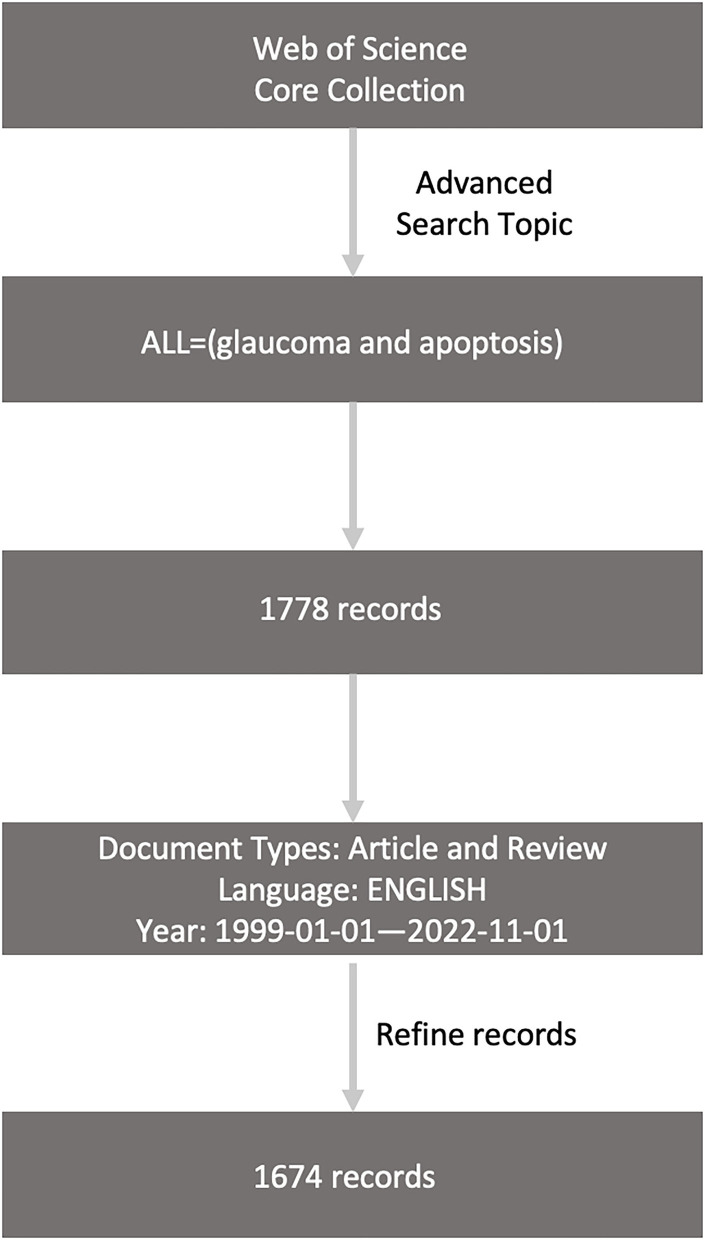
The data collection and retrieval strategy.

The data retrieval strategy and collection process for this study are shown in Figure 1. We collected the bibliographic data in the Web of Science Core Collection (WoSCC) and download the “Full record and cited references” of the literature in “plain text” format. We screened target literature based on: (1) search formula: ALL = (glaucoma and apoptosis); (2) Document type: “Article” and “Review”; (3) Publication date: 1 January 1999 to 1 November 2022; (4) Literature Language: English. The search was completed on 2 November 2022. Our initial search for research papers on glaucoma and apoptosis was first published in 1999, so we set our search to start in 1999. We ended up with 1,674 documents that met the criteria.

### Data analysis and network mapping

Bibliometrics is an efficient tool that helps researchers visualize the evolution of published literature. The analytical dimensions of bibliometrics include the year of publication, country and region, institution, journal, author, keywords, and co-citations. We imported the data from WoSCC into VOSviewer (version 1.6.18; https://www.vosviewer.com/downloavosviewer) and CiteSpace (version 5.8.R3; https://sourceforge.net/project/citespace/files/latest/do). CiteSpace and VOSviewer can be used to analyze the potential information contained in complex data, and present the structure, law, and distribution of information through visual means. Co-occurrence analysis refers to counting the frequency of occurrence of multiple phrases in the same article to determine their proximity, so as to obtain hot spots and future trends in the discipline. Co-citation analysis is used to discover the basic literature and knowledge in this research field (Romero and Portillo-Salido, 2019). The result provides information on the past, present, and future dimensions of the research field. In addition, we used Microsoft Office Excel 2021 to analyze trends in published articles over time.

In the visualization map below, each circle represents a node, and the diameter size of the circle represents how often the label appears in the co-occurrence analysis. The color of a circle is determined by the cluster of categories to which it belongs. The connection between nodes represents the association of the corresponding node, and the strength of the association between nodes is expressed in line width.

## Results

### Annual global publication outputs on glaucoma and apoptosis

**Figure 2:**
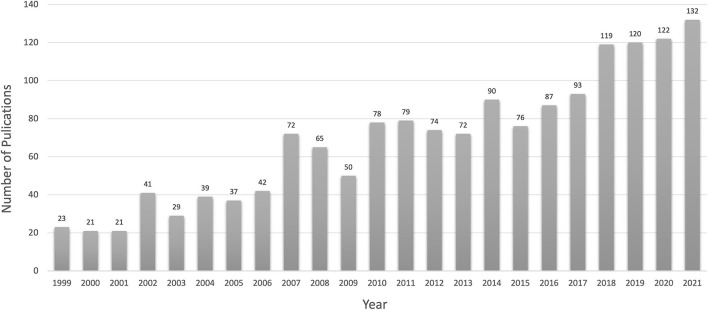
The number of articles published has grown steadily and peaked in 2021.

The annual publication trend of apoptosis in glaucoma is shown in Figure 2. From 1999 to 2022, the amount of literature in this field increased from 1999 (*n* = 23, 1.37%) to 2021 (*n* = 132, 7.89%), and peaked in 2021. As of November 2022, we believe that the number of publications in 2022 will increase, following previous years' trends.

### Co-authorship of countries/regions

**Figure 3:**
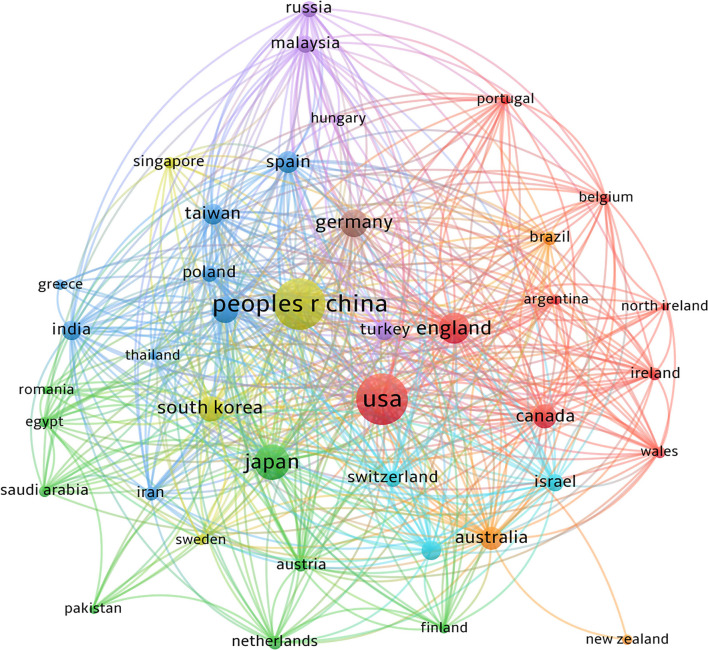
A total of 56 countries or regions have participated in the study of apoptosis in glaucoma. China and the United States are at the center of the study. The red cluster is centered in the United States. China's cooperation between countries and regions is relatively weak.

VOSviewer was used to analyze the authors' countries, regions, and partnerships, and the results are shown in Figure 3. Table 1 lists 10 countries with the highest number of publications. China published the most literature (482 documents and 7,217 citations), but the United States was the most influential country in this field (468 documents and 24,687 citations) in terms of overall citations. A network of coauthors from 40 countries or regions with more than three studies was divided into eight cluster groups with different color representations. The largest cluster (red) consisted of nine countries centered in the United States, the United Kingdom, and Canada.

**Table 1:** The top 10 productive countries/regions.

**Rank**	**Country**	**Documents**	**Citation**
1	China	482	7,217
2	USA	468	24,687
3	Japan	166	5,410
4	England	114	6,788
5	Germany	85	3,412
6	Italy	75	2,859
7	South	70	1,678
8	Korea	60	2,485
9	Canada	52	1,625
10	Australia	45	1,378

### Distribution of source journals and top 10 high-cited articles

Articles on glaucoma and apoptosis were published in 435 journals. Table 2 lists the top 10 journals with the most publications, accounting for 31.7% (531 documents and 1,674 citations) of all publications, and the distribution has a strong head effect. *Investigative Ophthalmology & Visual Science* was the most prolific journal (154 documents) and the most cited (9,011 citations), followed by *Experimental Eye Research* (80 documents). Although *Progress in Retinal and Eye Research* published only four studies, it was cited 4,183 times, indicating that the journal has a high impact factor.

**Table 2:** The top 10 journals for publications.

**Journals**	**Documents**	**Citation**	**IF (2022)**
Investigative ophthalmology and visual science	154	9,011	4.925
Experimental eye research	90	2,409	3.770
Molecular vision	60	1,598	2.711
Current eye research	52	981	2.555
Plos one	49	1,328	3.752
International journal of molecular science	28	240	6.208
Brain research	27	1,378	3.610
International journal of ophthalmology	26	154	1.645
Journal of glaucoma	24	1,019	2.290
Cell death and disease	21	783	9.685

A total of 114 articles were cited more than 100 times. Table 3 lists the top 10 cited literature. Among them, “Muller Cells in The Healthy and Diseased Retina” (Bringmann et al., 2006), published in *Progress in Retinal and Eye Research* in 2006, was the most cited literature (1,178 citations). The lowest number of citations overall was 327.

**Table 3:** The top 10 highest cited articles.

**Title**	**Journal**	**Citation**	**PY**
Muller cells in the healthy and diseased retina	Progress in retinal and eye research	1,178	2006
Neuronal death in glaucoma	Progress in retinal and eye research	582	1999
Preservatives in eyedrops: the good, the bad and the ugly	Progress in retinal and eye research	566	2010
The role of the reactive oxygen species and oxidative stress in the pathomechanism of the age-related ocular diseases and other pathologies of the anterior and posterior eye segments in adults	Oxidative medicine and cellular longevity	563	2016
The molecular basis of retinal ganglion cell death in glaucoma	Progress in retinal and eye research	549	2012
Para-inflammation in the aging retina	Progress in retinal and eye research	459	2009
Obstructed axonal transport of BDNF and its receptor TrkB in experimental glaucoma	Investigative ophthalmology and visual science	408	2000
The role of apoptosis in age-related macular degeneration	Archives of ophthalmology	398	2002
Unexpected low-dose toxicity of the universal solvent DMSO	Faseb journal	367	2014
Neuroprotection in relation to retinal ischemia and relevance to glaucoma	Survey of ophthalmology	327	1999

PY, Published Year.

### Distribution and co-authorship of institutions

The top 10 institutions with the highest number of publications are listed in Table 4. University of Wisconsin (48 documents and 2,754 citations) appears to be the most influential institution in the field, with the second-highest number of publications and the highest number of citations. Fudan University and Shanghai Jiao Tong University, two Chinese universities, have published more literature but have lower citations (526 citations and 522 citations, respectively).

**Table 4:** The top 10 productive institutions.

**Rank**	**Organization**	**Country**	**Documents**	**Citation**
1	Sun Yat Sen University	China	49	1,449
2	University Wisconsin	USA	48	2,754
3	Fudan University	China	44	526
4	Shanghai Jiao tong University	China	36	522
5	UCL	Britain	31	2,023
6	Johns Hopkins University	USA	23	2,317
7	University Calif San Diego	USA	22	1,185
8	University Oxford	Britain	22	2,572
9	University Texas	USA	22	1,084
10	Harvard University	USA	21	1,452

**Figure 4:**
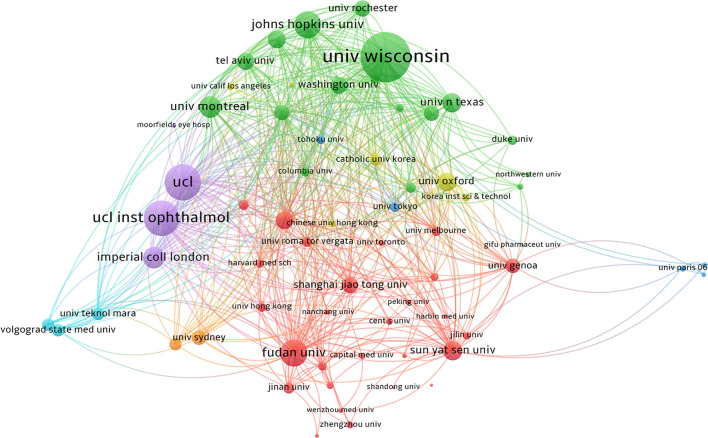
A total of 1,538 institutions participated in the study of apoptosis in glaucoma. The network of co-authors was divided into seven clusters of different colors. The green cluster is dominated by American institutions, and the University of Wisconsin had the greatest impact. The red cluster is dominated by Chinese institutions.

The network of coauthors from the research institutions is shown in Figure 4. Regardless of the green or red cluster, the vast majority of cooperation is limited to the country.

### Distribution and co-authorship of authors

**Figure 5:**
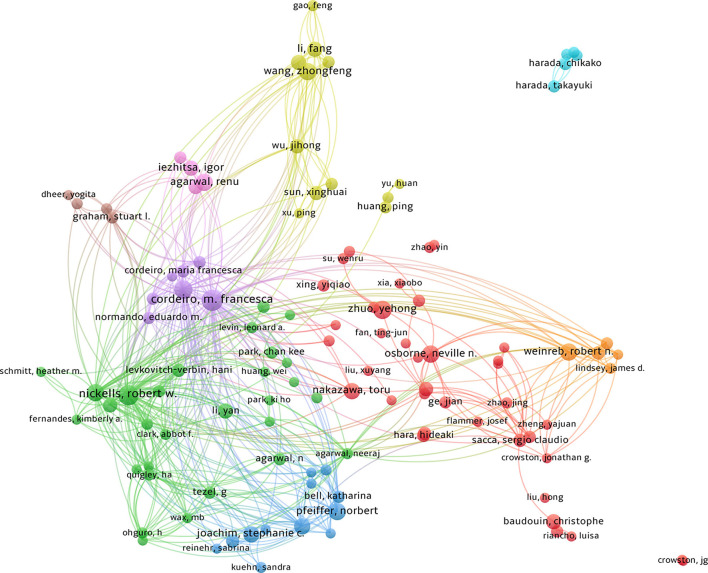
A total of 6,888 authors participated in the writing process. The Linlog/modular model was used for analysis. The weight is Total Link Strength. The size of the circle represents the amount of influence of the author. Inter-node connections represent author collaboration and the lines of connection between nodes represent the collaboration between authors.

The top five authors who published the most articles were all from England, the USA, and China. Dr. Cordeiro from Imperial College London has published 22 articles over the past 31 years, ranking as the top author in the field of apoptosis in glaucoma. Table 5 lists the top 10 authors with the highest number of published articles. Cordeiro, M. Francesca (22 documents and 1,254 citations) was the most prolific, followed by Nickells, Robert W. (20 documents and 996 citations). We set the threshold to five documents and screened a total of 182 authors who met the criteria; the results are shown in Figure 5. There are nine color classifications, of which the largest is the red cluster, composed of 38 authors centered around Osborne, Neville N, Zhuo, and Yehong.

**Table 5:** The top 10 productive authors.

**Rank**	**Author**	**Total publications**	**Citation**	**Avg citation**
1	Cordeiro, M. Francesca	22	1254	57
2	Nickells, Robert W.	20	996	50
3	Guo, Li	17	820	48
4	Zhuo, Yehong	17	701	41
5	Schlamp, Cassandra L.	16	771	48
6	Pfeiffer, Norbert	16	276	17
7	Wang, Zhongfeng	15	245	16
8	Weinreb, Robert N.	15	920	61
9	Agarwal, Renu	15	149	10
10	Osborne, Neville N.	15	2095	140

### Co-citation analysis of cited references

**Figure 6:**
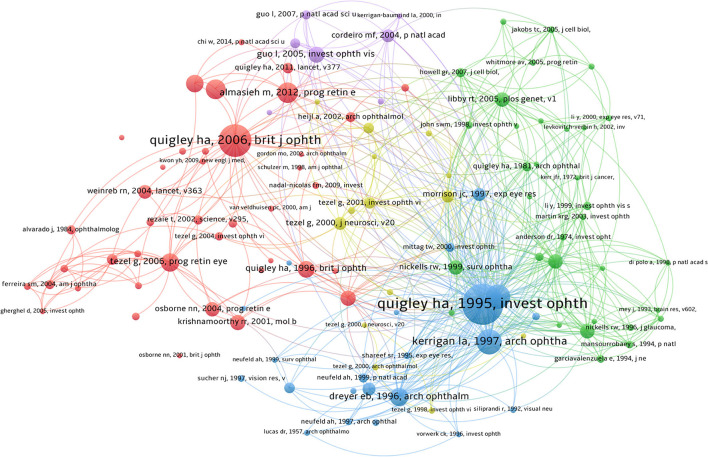
A total of 58,156 literature was cited from 1,674 retrieved literature. The two most cited articles in co-citation analysis were written by Quigley HA. The same color clusters indicate the documents are related or have some commonalities.

We listed the top 10 citations in Table 6, with citations ranging from 96 to 231. As shown in Figure 6, the paper titled “Retinal ganglion cell death in experimental glaucoma and after axotomy occurs by apoptosis” (Quigley et al., 1995), published in *Ophthalmology & Visual Science* in 1995, has the most citations (231 citations).

**Table 6:** The top 10 most co-cited references.

**Rank**	**First author**	**Year**	**Journal**	**Title**	**Citations**
1	Quigley HA	1995	Invest ophthalmol vis sci	Retinal ganglion cell death in experimental glaucoma and after axotomy occurs by apoptosis	231
2	Quigley HA	2006	British Journal of ophthalmology	The number of people with glaucoma worldwide in 2010 and 2020	185
3	Kerrigan LA	1997	Arch ophthalmol	TUNEL-positive ganglion cells in human primary open-angle glaucoma	141
4	Garcia Valenzuela E	1995	Exp eye res	Programmed cell death of retinal ganglion cells during experimental glaucoma	139
5	Almasieh M	2012	Prog retin eye res	The molecular basis of retinal ganglion cell death in glaucoma	118
6	Tham YC	2014	Ophthalmology	Assessment of iris surface features and their relationship with iris thickness in Asian eyes	114
7	Dreyer EB	1996	Archives of ophthalmology	Elevated glutamate levels in the vitreous body of humans and monkeys with glaucoma	105
8	Tezel G	2006	Prog retin eye res	Oxidative stress in glaucomatous neurodegeneration: mechanisms and consequences	102
9	Robert N Weinreb	2014	JAMA	The pathophysiology and treatment of glaucoma: a review	98
10	Quigley HA	1996	British journal of ophthalmology	Number of people with glaucoma worldwide	96

**Figure 7:**
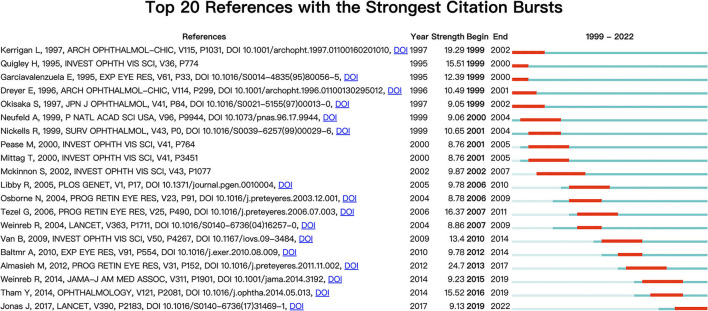
A citation burst is when the citation situation of a paper changes dramatically in a short period of time. The red line indicates the outbreak's duration, and the outbreak's intensity indicates the article's impact. The citation burst is made with CiteSpace.

Figure 7 shows the top 20 documents with the strongest citation outbreaks. “TUNEL-positive ganglion cells in human primary open-angle glaucoma” (Kerrigan et al., 1997), published in *Arch Ophthalmol* in 1997, had the highest impact at 19.29. The study proposes that apoptosis plays an important role in nerve cell death in primary open-angle glaucoma. “Global prevalence of glaucoma and projections of glaucoma burden through 2040: A systematic review and meta-analysis” (Tham et al., 2014), published in *Ophthalmology* in 2014, has the most citation outbreaks in recent years, with researchers predicting that the number of glaucoma cases will increase to more than 100 million by 2040. This poses a significant threat to human eye health.

### The co-occurrence analysis of the keywords

Keywords can indicate the topic of the publication; therefore, by analyzing keywords, we can understand emerging trends in research. We extracted and clustered the top 50 keywords in the study using VOSviewer (Table 7). The keywords were glaucoma (526 times), apoptosis (384 times), retinal ganglion cells (341 times), neuroprotection (201 times), and oxidative stress (122 times).

**Table 7:** The top 50 Keywords.

**Keywords**	**Counts**	**Rank**	**Keywords**	**Counts**	**Rank**
Glaucoma	526	1	Neuroinflammation	17	26
Apoptosis	384	2	Optic nerve injury	17	27
Retinal ganglion cells	365	3	Hypoxia	16	28
Neuroprotection	201	4	Reactive oxygen species	16	29
Retina	123	5	Age-related macular degeneration	15	30
Oxidative stress	122	6	Cell death	15	31
Intraocular pressure	55	7	Ischemia	15	32
Neurodegeneration	54	8	Benzalkonium chloride	14	33
Optic nerve	49	9	Caspase	14	34
Autophagy	41	10	Cornea	14	35
Trabecular meshwork	41	11	Nitric oxide	14	36
Excitotoxicity	39	12	Primary open angle glaucoma	14	37
Mitochondria	36	13	Aqueous humor	13	38
Inflammation	35	14	Ganglion cell	13	39
Diabetic retinopathy	23	15	Resveratrol	13	40
Glutamate	23	16	Alzheimer's disease	12	41
Microglia	21	17	Citicoline	11	42
Rat	21	18	Eye	11	43
Retinal degeneration	21	19	Imaging	11	44
Optic neuropathy	20	20	akt	10	45
Ocular hypertension	19	21	Antioxidants	10	46
Nmda	18	22	bcl-2	10	47
Optic nerve crush	18	23	Caspase-3	10	48
Primary open-angle glaucoma	18	24	Endoplasmic reticulum stress	10	49
rgc-5	18	25	Wound healing	10	50

### Most frequently in the author's keywords

**Figure 8:**
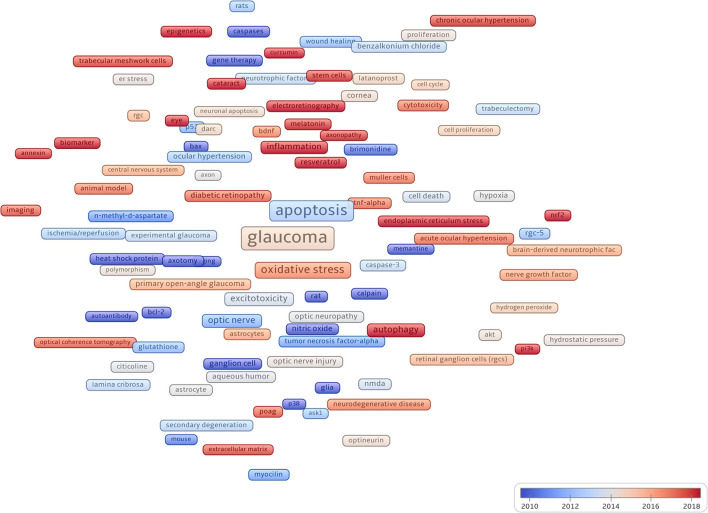
Node square sizes indicate how often keywords appear, cooler colors indicate earlier occurrences of keywords, and warmer colors indicate later occurrences.

We color-coded keywords according to the average year of occurrence (AAY) in the literature in the field, then explored the evolution trend of keywords over time, and visualized them in Figure 8. Recent keywords included mitophagy (AAY:2022.17), endoplasmic reticulum stress (AAY:2019.44), autophagy (AAY:2018.03), melatonin (AAY:2017.83), proteomics (AAY:2016.67), and epigenetics (AAY:2016.15).

**Figure 9:**
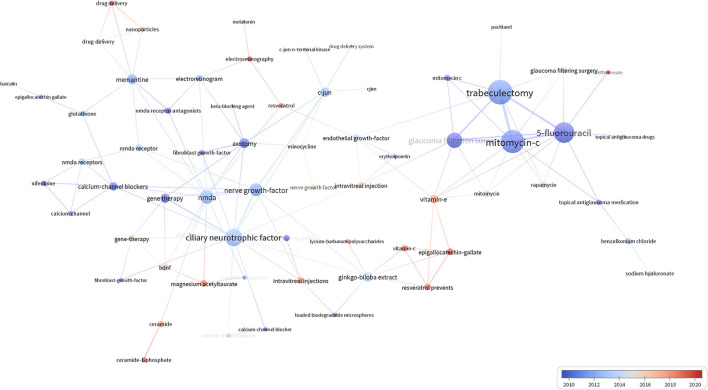
The width of the line between the nodes indicates the strength of the association between the keywords.

Concerning glaucoma treatment progress, we listed the top 60 keywords occurring most frequently in Table 8, covering glaucoma drug treatment, surgical treatment, and eye examination. Figure 9 is a visualization of Table 8. The most frequent keyword was *N-Methyl-D-aspartate* (NMDA) receptor antagonists (59 times). NMDA-induced excitatory toxicity plays an important role in glaucoma-induced RGC death and the lack of NMDA receptor subunits in experiments can reduce the excitotoxic effect of NMDA, thereby protecting RGCs in mice (Hayashi et al., 2021). A classic treatment for glaucoma is trabeculectomy, which appeared 39 times. In addition, trabeculectomy in combination with antimetabolites, such as mitomycin-c (31 times) or 5-fluorouracil (19 times), has long been known to improve prognosis. We could see a close connection between the three keywords (Figure 9). In summary, we obtained an overview of glaucoma treatment using keyword analysis.

**Table 8:** The top 60 keywords related to glaucoma treatment.

**Keywords**	**Counts**	**Rank**	**Keywords**	**Counts**	**Rank**
nmda receptor antagonists	59	1	Astaxanthin	4	31
Trabeculectomy	39	2	Beta-blockers	4	32
Mitomycin-c	31	3	Cobalt chloride	4	33
Benzalkonium chloride	27	4	Fibroblast growth-factor	4	34
Ciliary neurotrophic factor	27	5	Penetrating keratoplasty	4	35
Optical coherence tomography	25	6	Peripheral-nerve grafts	4	36
Endothelial growth-factor	24	7	Pilocarpine	4	37
C-jun	23	8	Rapamycin	4	38
Nerve growth-factor	23	9	Vitamin-c	4	39
Axotomy	21	10	Vitamin-e	4	40
Drug delivery	21	11	Statins	4	41
5-fluorouracil	19	12	Baicalin	3	42
Bdnf	19	13	Puerarin	3	43
Electroretinogram	16	14	Quercetin	3	44
Glaucoma filtration surgery	16	15	Sodium hyaluronate	3	45
Resveratrol	15	16	Valproic acid	3	46
Memantine	14	17	Aldosterone	2	47
Ginkgo-biloba extract	12	18	Aloe-emodin	2	48
Glutathione	12	19	Arbutin	2	49
Gene therapy	10	20	Calcium channel blocker	2	50
Melatonin	10	21	Loaded biodegradable microspheres	2	51
Epigallocatechin gallate	9	22	Methotrexate	2	52
Intravitreal injection	8	23	Methylprednisolone	2	53
Ceramide	7	24	Nifedipine	2	54
Magnesium acetyltaurate	7	25	Paclitaxel	2	55
Erythropoietin	6	26	Ripasudil	2	56
Minocycline	6	27	Rosiglitazone	2	57
Nanoparticles	6	28	Scutellarin	2	58
Scanning laser ophthalmoscope	6	29	Selective laser trabeculoplasty	2	59
Topical antiglaucoma medication	6	30	Lutein	2	60

## Discussion

In this current study, we used bibliometric tools to visualize and analyze current publications in the area of apoptosis in glaucoma and found that the publications on apoptosis in glaucoma increased over time. In addition, authors and universities in the US and China may be leading research in this field. It should also be noted that a series of advanced research results, technologies, and treatments for glaucoma, such as the discovery of key regulatory mechanisms of RGC apoptosis, are emerging and will provide strategies for glaucoma treatment.

In the current study, we conclude the increasing trend of publications over the years indicates the importance and progress of research in the field of apoptosis in glaucoma. Presently, China, the US, and Japan are the top three countries with the highest number of articles, implying that they are the research centers in this field. The most cited research is Dr. Quigley's (John Hopkins Hospital), “Retinal ganglion cell death in experimental glaucoma and after axotomy occurs by apoptosis (Quigley et al., 1995), which was cited 231 times. It indicated that RGCs die due to apoptosis after axotomy injury and glaucoma. This provided preliminary evidence that apoptosis is the mode of RGC death and may be triggered by excitotoxins, which is important for designing strategies to protect RGCs. The journal *Ophthalmology & Visual Science* published the most articles on apoptosis in glaucoma. *Cell Death & Disease* was the journal with the highest impact factor (IF), IF 2021 = 9.685. Prof. Bringmann published a review “Muller cells in the healthy and diseased retina” (Bringmann et al., 2006) in *Progress In Retinal and Eye Research*, which is one of the top five journals (IF > 10) and is cited 1,178 times. It indicated that retinal diseases such as glaucoma are associated with Muller cell reactivation. Under normal conditions, Muller cells maintain retinal neurons by releasing neurotrophic factors and deactivating excitotoxins. After injury or disease, Muller cells may accelerate neuronal death, as they can be dysregulated leading to a disturbance in glutamate metabolism in the retina, ultimately causing neuronal cell death. A deeper understanding of Muller cell reactivation and targeting may be a useful therapeutic strategy to protect retinal neurons against apoptosis.

Furthermore, we used co-occurrence cluster analysis to produce and analyze the network graph of keywords found in studies on apoptosis in glaucoma. Based on the analysis of keyword frequencies, research hotspots could be identified. Among the top 50 most common keywords were: caspase, akt, bcl-2, and nitric oxide. The keyword results showed that research trends in glaucoma are diversifying, which is not only restricted to ophthalmology but also included progress in cell biology, biochemistry, and genetics. Based on keyword co-occurrence analysis, changes in research trends could be visualized. Initially, keywords including glaucoma, apoptosis, retinal ganglion cells, neuroprotection, and oxidative stress appeared earlier. In the analysis of frequencies and centralities of the keywords, drugs such as “benzalkonium chloride,” “resveratrol,” and “citicoline,” and keywords related to gene research, such as “caspase,” “akt,” and “bcl-2,” appeared earlier. These results suggest that more research on pathological mechanisms and treatment methods was carried out. In addition, after 2016, keywords such as mitophagy, endoplasmic reticulum stress, gliosis, inflammation, autophagy, melatonin, annexin, cytotoxicity, proteomics, and epigenetics were mostly retrieved and showed persistence in recent years, suggesting that this field is the next key direction and are hotspot in studies on apoptosis in glaucoma.

High IOP is the primary risk factor for glaucoma (Cheng et al., 2018). However, IOP measurement alone is not the sole diagnostic standard for glaucoma (Gedde et al., 2021; Steiner et al., 2022). Patients with any symptoms of glaucoma will progress to irreversible RGC loss and optic nerve degeneration (Wang et al., 2017b; Donahue et al., 2021). By analyzing emerging research in glaucoma treatment, some keywords related to examination and treatment have been developed in recent years. The most frequent keyword is NMDA receptor antagonists. NMDA is the most frequent and important excitatory acid in the nervous system, which induces excitatory toxicity and plays an important role in glaucoma-induced RGC death (Hayashi et al., 2021). In experimental glaucoma models, lack of NMDA receptors or NMDA inhibition can reduce the excitotoxic effects of NMDA, thereby protecting RGCs in mice (Hayashi et al., 2021; Sato et al., 2021). As a classic treatment for glaucoma (Olawoye and Etya'ale, 2021; Philippin et al., 2021), trabeculectomy appears 39 times, ranking as the second most frequent treatment keyword. In addition, trabeculectomy in combination with antimetabolites such as mitomycin-c (top three treatment keyword) or 5-fluorouracil (top 12 treatment keyword) has long been known to improve the prognosis for glaucoma treatment (Theventhiran et al., 2021; Al-Mugheiry et al., 2022; Yang et al., 2022). Ophthalmoscopy is a common technology used to detect the optic nerve head and neuronal loss (Coote, 1857; Quigley and Anderson, 1977). We also found that optical coherence tomography (OCT) appeared in 2011 and ranked among the top six treatment keywords, indicating that it is key in research on apoptosis in glaucoma due to its relationship with other keywords. OCT can provide more detailed information about the loss of the optic nerve head and neurons quantitatively. However, in the early stages of glaucoma it is difficult to detect neuronal injuries, which would be permanent (Shao et al., 2022; Zhou et al., 2022). Therefore, early diagnosis and intervention to delay the progress of glaucoma and achieve a better prognosis are urgently needed. Scanning laser ophthalmoscopy, Doppler OCT, and other modified OCTs can be combined to examine capillary morphology and sensitively detect blood flow in the retina in early glaucoma (Aumann et al., 2019; Burns et al., 2019). However, at present, the only effective way to delay glaucoma progression is to reduce IOP using eye drops, surgery, or laser procedures (Schehlein and Robin, 2019; Rolim-De-Moura et al., 2022). Prostaglandins are the most common medication used to lower IOP by accelerating aqueous drainage through the uveal sclera (Jansook and Loftsson, 2022). Other drugs, such as α-adrenergic agonists, β-adrenergic blockers, and carbonic anhydrase inhibitors are second-line drugs that are ineffective in reducing IOP and have side effects (Lu et al., 2017; Nocentini and Supuran, 2019; Stoner et al., 2022). Surgical or laser procedures are recommended when medication cannot sufficiently lower IOP (Schehlein and Robin, 2019; Rolim-De-Moura et al., 2022). In recent years, advances in surgery, drug delivery, and gene therapy have led to a new field of glaucoma treatment (Wan et al., 2021; Tawfik et al., 2022). However, the impaired visual function still becomes serious after lowering the IOP in some patients (Jayanetti et al., 2020; Baudouin et al., 2021) as RGCs and the optic nerve irreversibly degenerate (Jayanetti et al., 2020; Baudouin et al., 2021). Therefore, research on the pathological mechanisms and development of neuroprotective treatments are still hotspots in glaucoma research.

### Limitations

Our literature retrieval work was conducted from 1 January 1999 to 1 November 2022. Therefore, articles published after 2 November 2022 with advanced research results that met the search criteria were not included, making the number of articles in our database less than the total number today. In addition, we only included articles and reviews as we aimed to control the quality of publications as much as possible. Other types of publications, such as case reports, meta-analyses, and books were excluded, even though, they may contain valuable information. Apoptosis in glaucoma encompasses several research topics. Although we made our best effort to retrieve more relevant publications, we may have overlooked important research and results using our current retrieval formula considering the limitations of bibliometric tools such as CiteSpace and VOSviewer.

## Conclusion

This study aimed to determine the current and future trends and hotspots of apoptosis in glaucoma. We used bibliometric tools to visualize and analyze publications in this field and found that publications on apoptosis in glaucoma increased over time. It is an emerging series of advanced research involving technologies and treatments for glaucoma, such as the discovery of key regulatory mechanisms of RGC apoptosis, which will provide precise strategies for glaucoma treatment. In summary, this study can deepen our understanding of the trends and frontiers of apoptosis in glaucoma and provide guidelines for future research and treatment.

## Data availability statement

The raw data supporting the conclusions of this article will be made available by the authors, without undue reservation.

## Author contributions

S-CW designed the experiments, interpreted the data, and revised the manuscript. J-HZ and M-JW performed the experiments, prepared the figures, and wrote the manuscript. Y-TT and JL helped to perform the experiments and prepared the figures. All authors read, discussed, and approved the final manuscript.
